# Hydrophobic mismatch and sequence specificity compete when transmembrane helix-helix interactions are measured with the TOXCAT assay

**DOI:** 10.3389/fchem.2022.1049310

**Published:** 2022-11-28

**Authors:** Nadja Hellmann, Dirk Schneider

**Affiliations:** ^1^ Department Chemie—Biochemie, Johannes Gutenberg-Universität Mainz, Mainz, Germany; ^2^ Institut für Molekulare Physiologie, Johannes Gutenberg-Universität Mainz, Mainz, Germany

**Keywords:** TOXCAT, GpA, dimerization, biological assay, hydrophobic mismatch, transmembrane helix, protein folding

## Abstract

Genetic assays capable of measuring the propensity of transmembrane helices to oligomerize within the cytoplasmic membrane of the bacterium *E. coli* are frequently used when sequence-specificity in transmembrane helix-helix interactions is investigated. In the present study, dimerization of the well-investigated wild-type and G83I-mutated transmembrane helix of the human glycophorin A protein was studied. Gradual prolongation of the transmembrane helix at the C-terminus with Leu residues lead to pronounced changes in the dimerization propensity when measured with the TOXCAT assay. Thus, besides sequence specificity, hydrophobic mismatch between the hydrophobic core of a studied transmembrane helix and the *E. coli* membrane can impact the oligomerization propensity of a transmembrane helix. This suggests that the results of genetic assays aiming at determining interactions of heterologous transmembrane helices within the *E. coli* membrane do not necessarily solely reflect sequence specificity in transmembrane helix-helix interactions, but might be additionally modulated by topological and structural effects caused by hydrophobic mismatch.

## Introduction

Defined interactions of individual transmembrane (TM) helices are key for proper folding and functioning of α-helical membrane proteins. Ideally, already individual TM helices are stable within a membrane, interact and form higher-order oligomeric structures (Popot and Engelman, 1990). While in multispan TM proteins the folding pathway can be far more complex, potentially involving a variety of folding intermediates (Hong et al., 2022), in case of single-span TM proteins the existence of stable individual TM helices is likely. In fact, single-span TM proteins cover almost half of the whole human membrane proteome (Worch et al., 2010), and many of these have already been shown to interact with each other and to from higher-order oligomeric structures (Finger et al., 2009; Kirrbach et al., 2013).

The first method to study the interaction of individually stable TM helices within the *E. coli* cytoplasmic membrane was developed more than 20 years ago (Langosch et al., 1996). In this method, the ToxR assay, the TM helix of interest is fused to the DNA-binding domain of the ToxR transcription activator of *Vibrio cholera*, and TM helix-helix interactions result in formation of a DNA-binding domain dimer. As only the dimer can bind to the *ctx* promoter/operator region, TM helix dimerization in the end controls expression of the *lacZ* reporter gene, which has been placed under control of the *ctx* promoter. The reporter gene activity is believed to directly reflect the oligomerization propensity of a given TM helix. At the TM helix’ C-terminus, the *E. coli* MalE protein is genetically fused to facilitate membrane integration of the fusion protein (Kolmar et al., 1995) and to enable straightforward determination of membrane integration and the TM topology of the expressed fusion protein (Langosch et al., 1996; Russ and Engelman, 1999). The original ToxR-assay was modified later by the Engelman group (Russ and Engelman, 1999), and these days, the TOXCAT assay is one of the most frequently applied genetic systems to study TM helix-helix interactions. Similar assays, which allow measuring homo- as well as hetero-dimerization of TM domains, were developed in recent years (Schneider and Engelman, 2003; Lindner and Langosch, 2006; Lindner et al., 2007; Su and Berger, 2013). Meanwhile, such *in vivo* assays have become standard tools to study TM helix-helix interactions within the *E. coli* inner membrane, and even the energetics of TM helix-helix interactions have been estimated using such genetic systems (Finger et al., 2006; Duong et al., 2007; Prodöhl et al., 2007). However, in some studies, where sequence specificity in dimerization has been addressed based on such genetic assay, it has been observed that the length of the studied TM helix has an impact on the assay outcome (Schneider and Engelman, 2003; Li et al., 2004; Zhu et al., 2010; Grau et al., 2017). These observations now raise the question as to what additional factors affect an apparent dimerization propensity since sequence specificity in TM helix oligomerization *per se* should not be affected by the helix length.

In the present study, we have systematically analyzed the impact of the helix length on a TM helix dimerization propensity within the *E. coli* inner membrane using the TM helix of the human glycophorin A (GpA) protein as a model. Already more than 30 years ago it has been observed that the human GpA TM helix forms SDS-stable dimers in solution (Bormann et al., 1989). Based on a subsequent rigorous SDS-gel analysis of the GpA dimerization propensity, the seven amino acid motif LIxxGVxxGVxxT has been identified to be crucial for dimerization of the GpA TM helix (Lemmon et al., 1992). Critical involvement of these residues in dimer formation was later confirmed by the NMR structures of the GpA TM helix dimer in detergent and lipid bilayers (MacKenzie et al., 1997; Smith et al., 2001; Smith et al., 2002; Mineev et al., 2011) as well as by genetic systems (Brosig and Langosch, 1998; Russ and Engelman, 2000; Duong et al., 2007). However, detailed studies in various detergents and detergent-like environments have also indicated that the actual interaction propensity of the GpA TM domain depends on the chemical nature of the detergent (Fisher et al., 1999; Fisher et al., 2003; Fleming et al., 2004; Anbazhagan et al., 2010a; Stangl et al., 2012; Stangl et al., 2014). In fact, the structure of GpA in a detergent micelle is somewhat different compared to the one obtained in a lipidic environment (MacKenzie et al., 1997; Mineev et al., 2011; Trenker et al., 2015). The interaction mediated by the GpA dimerization motif also strongly depends on membrane properties (Anbazhagan and Schneider, 2010; Anbazhagan et al., 2010b; Hong and Bowie, 2011). These observations indicate that global membrane properties might counteract or enhance dimerization, and by this modulate sequence-specificity in TM helix dimerization. Molecular details of this modulation are largely enigmatic, yet it appears that sequence-specific interaction of the GpA TM helix peptide is optimal under hydrophobic matching conditions*,* i.e., if the thickness of a hydrophobic bilayer core matches approximately the hydrophobic thickness of the TM helix dimer (Orzaez et al., 2005; Anbazhagan and Schneider, 2010). If the TM helix is longer than that (positive hydrophobic mismatch) or shorter (negative hydrophobic mismatch), a number of structural adjustments are possible, as summarized e.g., in (Killian, 1998).

We have systematically prolonged the length of the GpA TM helix on the C-terminal end with Leu residues and analyzed the impact on helix dimerization within the *E. coli* membrane using the TOXCAT assay. The results indicate that the helix length significantly affects the interaction propensities determined with this assay. The observations strongly suggest that mismatch between the length of the hydrophobic TM and the hydrophobic thickness of the *E. coli* inner membrane can severely affect the measured degree of TM helix oligomerization. Consequently, results obtained with genetic systems must be interpreted with great caution.

## Materials and methods

### TOXCAT assay

**FIGURE 1:**
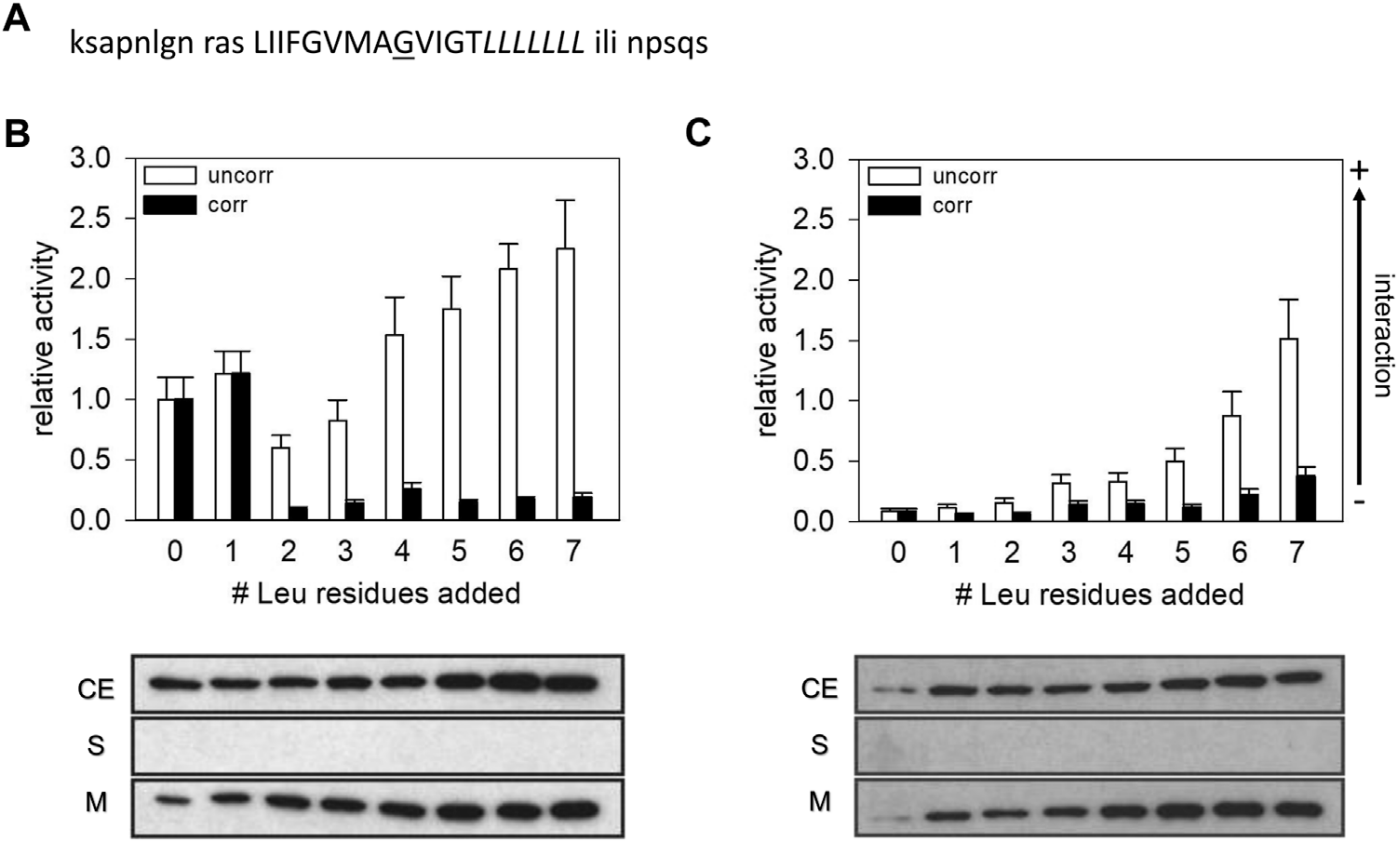
CAT activities measured with the construct expressing the GpA13 wt and GpA_G83I TM domain. **(A)** Sequence of the GpA TM domain expressed from the TOXCAT plasmid pccGpA wt and G83I. The Leu residues indicated in italics were successively added at the GpA TM helix’ C-terminus. The residue G83, which was mutated to Ile, is underlined. Residues, flanking the TM helix and which were introduced by the plasmids, are shown in low-case letters. **(B,C)** CAT activities of GpA wt **(B)** and GpA G83I **(C)** measured with the TOXCAT system. The measured activities were normalized to the activity of the wt GpA13 fusion protein. White bars: uncorrected data. Black bars: Activities corrected for differences in protein expression levels. Lower panel: Expression level and subcellular localization of the fusion proteins. Samples from *E. coli* cellular extract (CE), soluble protein fractions (S) and membranes (M) were analyzed *via* Western blots using an anti-MalE antibody.

Homodimerization of the TM domains was measured with the TOXCAT system (Russ and Engelman, 1999). The construction of the plasmid pccKan and of the chimera of GpA13 has been described (Russ and Engelman, 1999). To create plasmids expressing prolonged GpA TM regions (compare Figure 1), synthetic oligonucleotide cassettes (from Eurofins, Ebersberg, Germany) were ligated into the *Nhe*I/*Bam*HI restriction digested plasmid pccKan, resulting in the generation of an open reading frame, which codes for a chimeric protein that is N-terminally fused to the ToxR DNA-binding domain and C-terminally to the MalE domain of *E. coli*. The correct insertion of the DNA cassettes was checked by DNA sequencing.

For the TOXCAT measurements, plasmids were transformed into *E. coli* NT326 (Treptow and Shuman, 1985). Cells were grown in LB medium in presence of antibiotics at 37°C overnight and diluted in the same medium to OD_600_ = 0.1 the following morning. Cells were harvested at OD_600_ = 0.6 and chloramphenicol acetyl transferase (CAT) activities were measured as described in detail in (Sulistijo et al., 2003; Finger et al., 2009). The interaction propensity of the individual TM domains is presented as the mean ± standard deviation of the CAT activities measured with at least three independent clones, each measured three times.

In order to estimate the expression level of the fusion protein, Western blot analysis of whole lysates was performed using an anti-MalE antibody (New England Biolabs, Frankfurt, Germany). The band intensities [measured with the program ImageJ (Schneider et al., 2012)] of the various constructs were normalized to the band intensity of the reference construct GpA13 to yield relative amounts, and the measured CAT activities was divided by the square of the relative amount to obtain activities normalized to the amount of dimeric fusion protein (Duong et al., 2007). Each single plasmid used for the TOXCAT measurements was tested for proper integration of the encoded chimeric protein into the *E. coli* inner membrane *via* NaOH extraction of lysozyme treated cells, as described in detail in (Cymer et al., 2013). In order to determine the orientation of the fusion protein in the *E. coli* membrane, plasmids were transformed into *E. coli* NT326 cells, which are *malE* deficient (Treptow and Shuman, 1985), and growth on M9 medium with maltose as the only carbohydrate source was monitored. Since the cells are only able to complement the absence of endogenous MalE if the MalE domain of the chimeric proteins is located in the periplasm, proper growth indicates a correct topology of the inserted protein.

### Structure predictions

Modelling of the monomeric form of the construct was performed employing the software FMAP (Lomize et al., 2011), based on the subroutine “membrane protein.” Here, the lipid bilayer cannot be chosen, but is defined corresponding to a DOPC bilayer with a hydrophobic thickness of 28.8 Å. The dimer structure was modelled using TMDOCK (Lomize and Pogozheva, 2017), which in fact uses FMAP to first define the TM helix of the monomer, and subsequently calculates models for the dimeric structure. From the models suggested by TMDOCK, the one with the highest dimerization energy was selected. The position of this model within a Gram-negative inner membrane (as representative for the *E. coli* inner membrane) was calculated employing the PP3 software (Lomize et al., 2022).

## Results and discussion

### Dimerization propensity of GpA-PolyLeu transmembrane domains

To systematically monitor the influence of the TM helix length on a sequence-specific TM helix-helix interaction determined with a bacterial assay, we decided to monitor homo-oligomerization of the well-characterized GpA TM helix as a model. GpA TM helix dimerization within the *E. coli* inner membrane has been studied using several genetic systems (Brosig and Langosch, 1998; Russ and Engelman, 2000; Schneider and Engelman, 2003; Duong et al., 2007), including the well-established TOXCAT system (Russ and Engelman, 1999). A systematic analysis of integrin TM helix interactions has indicated that the register of an interaction-mediating GxxxG-motif with respect to the DNA-binding domain of the reporter system is important and might influence the actual strength of a measured interaction propensity when measured with a genetic system (Schneider and Engelman, 2004a; Li et al., 2004). Thus, to maintain the position of the ToxR DNA-binding domain with respect to the interacting surface of the GpA TM helix, Leu residues were introduced at the C-terminal TM helix end, between the GpA TM and the MalE domain. With this, the 13 amino acids long GpA TM sequence, which contains the complete motif crucial for GpA dimerization and which has been identified as the smallest construct able to form a stable TM dimer in the ToxR-based assay (Langosch et al., 1996; Russ and Engelman, 1999), was kept constant and remained fused directly to the ToxR DNA-binding domain (Figure 1A). Based on the NMR structure of the GpA TM dimer and on several mutational studies, the residues following Thr87 in the GpA TM sequence are not sufficiently close to interact, and thus are not involved in forming and/or stabilizing the GpA TM domain contact interface (Lemmon et al., 1992; MacKenzie et al., 1997). Thus, the addition of up to seven Leu residues at the C-terminus of the TM after Thr87 should not result in changes in the dimerization propensity *per se*. The resulting and here further analyzed chimeric proteins had a presumed total TM helix length between 13 and 20 amino acids.

As can be seen in Figure 1B, the CAT activity of the GpA wt TM construct significantly decreased when the helix length was increased to 15 a but thereafter steeply increased again when further Leu residues were added to the C-terminus of the TM helix. When seven Leu residues were added (GpA13L_7_), the CAT activity was almost 2.5-fold higher compared to the GpA13 construct. However, the measured CAT activity is linearly related to the overall amount of the dimer formed, which in turn is proportional to the square of the amount of monomeric total construct per cell. Therefore, we determined the relative contents of the expressed fusion proteins in the expression strains as well as the subcellular localization of the chimeric proteins by Western blot analysis (Figure 1B). In all cases, the expressed fusion proteins were exclusively located within the *E. coli* cytoplasmic membrane. However, while the expression levels of the GpA13 and the GpA13L_1_ constructs were rather similar, the amount of protein increased gradually when the number of Leu was increased from two to six (GpA13L_2_ to GpA13L_6_). Under the assumption that the fraction of dimers is low, the number of monomers can be approximated by the total amount of expressed GpA constructs. Thus, the CAT activities were corrected for the different expression levels by division of the squared relative band intensity obtained from the Western blots (Duong et al., 2007). This leads to a drastic change in the pattern: the corrected CAT activities indicate that adding one Leu does not alter the dimerization propensity much, but adding one further Leu leads to a drastic decrease compared to the GpA13 protein, which stayed more or less constant upon addition of further Leu residues.

This quite clear effect on the CAT activity upon increasing the TM length of GpA by more than one Leu was rather unexpected. Since the sequence is prolongated at the C-terminal helix end, an effect on the interaction between the ToxR-domain and the promoter seems unlikely. Thus, either the spatial arrangement of the dimer was altered, leading to ToxR-dimers that are not fully functional, or the dimerization propensity was altered due to effects, which stabilize the monomeric state compared to the dimeric one.

To further evaluate the interaction propensity of the Leu-prolonged GpA TM domain, we next investigated the dimerization behavior of the G83I mutated GpA TM domain (GpA13_G83I). In several studies it has been shown that Gly83 is key for GpA TM helix dimerization, and replacement of Gly83 by Ile strongly reduces dimerization of the TM domain due to steric hindrances (Lemmon et al., 1992; Russ and Engelman, 1999; Fleming and Engelman, 2001; Schneider and Engelman, 2004b).

**FIGURE 2:**
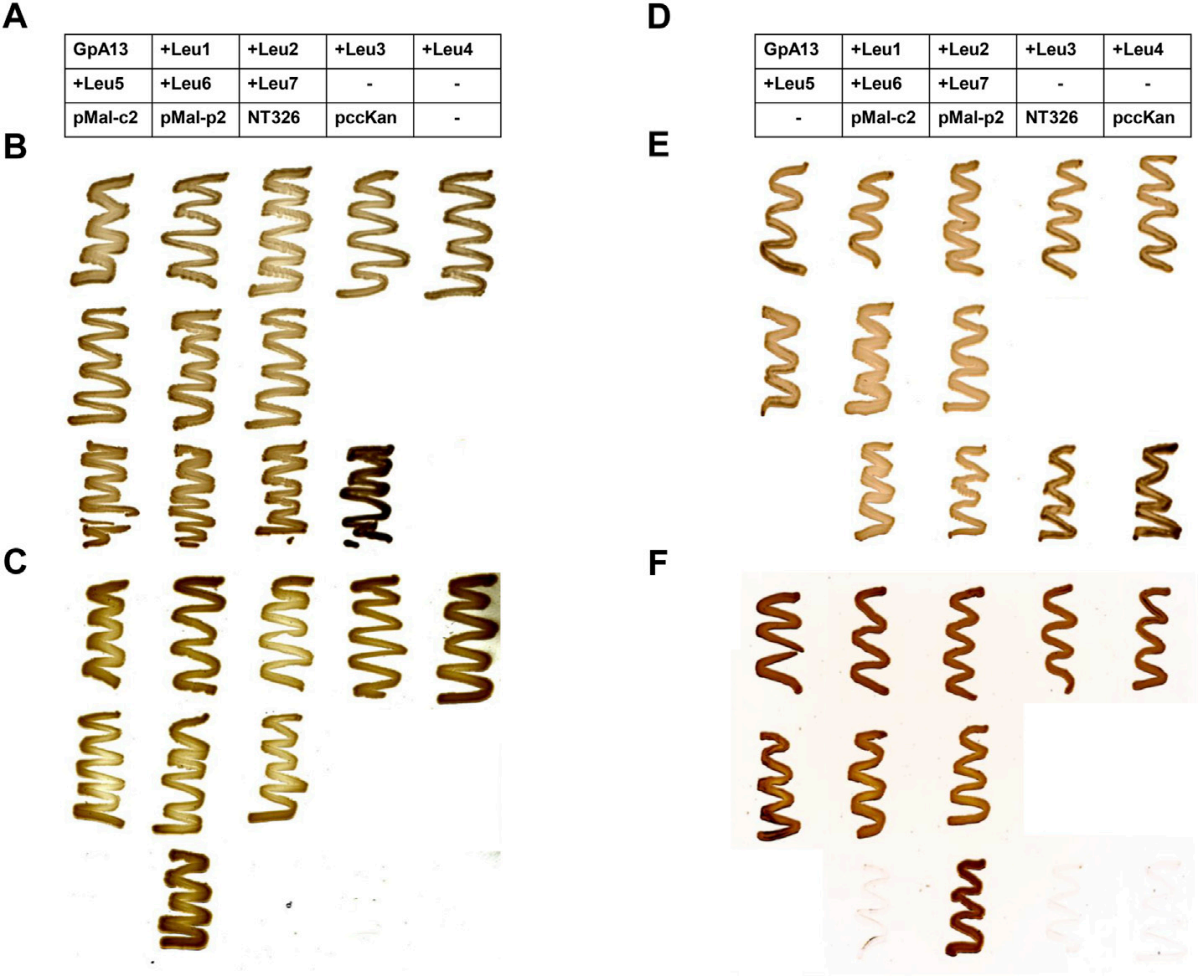
*malE* complementation assay. In order to test the orientation of the ToxR (TM) MalE chimeric proteins, MalE-deficient *E. coli* NT326 cells were transformed with plasmids encoding the chimeric proteins and cultivated on M9 minimal medium supplemented with either glucose **(B,E)** or maltose **(C,F)**. MalE-deficient cells can grow on maltose as the sole carbohydrate source exclusively when the MalE domain of the expressed chimeric protein is located within the *E. coli* periplasm. In contrast, when glucose is offered as a carbohydrate source, all cells are expected to grow. Cytoplasmic MalE expression from the plasmid pMal-c2, as well as the MalE-deficient strain NT326, and NT326 cells transformed with the pccKan plasmid used for cloning and construction of GpA TM helix-expressing chimeric proteins served as negative controls. These cells should not grow on maltose-containing medium. In contrast, periplasmic MalE expression from the plasmid pMal-p2 was expected to complement for the *malE* deficiency of NT326 cells, and thus serves as a positive control. Expression of all chimeric proteins resulted in complementation of the NT326 growth defect on maltose-containing medium, and thus all encoded proteins were expressed with the correct TM topology, i.e., the MalE domain located within the *E. coli* periplasm. **(A–C)**: GpA wt; **(D,E)**: GpA G83I. The tables in **(A,D)** describe the location of *E. coli* NT326 cell transformed with a respective plasmid on the plate. When columns of the table are empty (marked with an “−”), no cells were streaked out on the respective positions on the plate.

As has been shown many times before, the interaction propensity of the GpA13_G83I TM domain (Figure 1C) is dramatically lower when compared to the GpA13 wt construct (Figure 1B). Yet, the measured CAT activities continuously increase with increasing TM helix length. Again, after correction for differences in the expression levels, the pattern drastically altered, and only small (compared to the error) differences in dimerization propensity are observed upon prolongation of the TM helix (Figure 1C). Noteworthy, both for the wt and the mutant constructs, expression of all analyzed chimeric proteins resulted in complementation of the *E. coli* NT326 growth defect (Figure 2), which was caused by deletion of the endogenous *malE* gene (Treptow and Shuman, 1985). Thus, the chimeric proteins had the correct TM topology, with the fused MalE domain located in the *E. coli* periplasm.

### Hydrophobic mismatch vs. sequence-specificity

**FIGURE 3:**
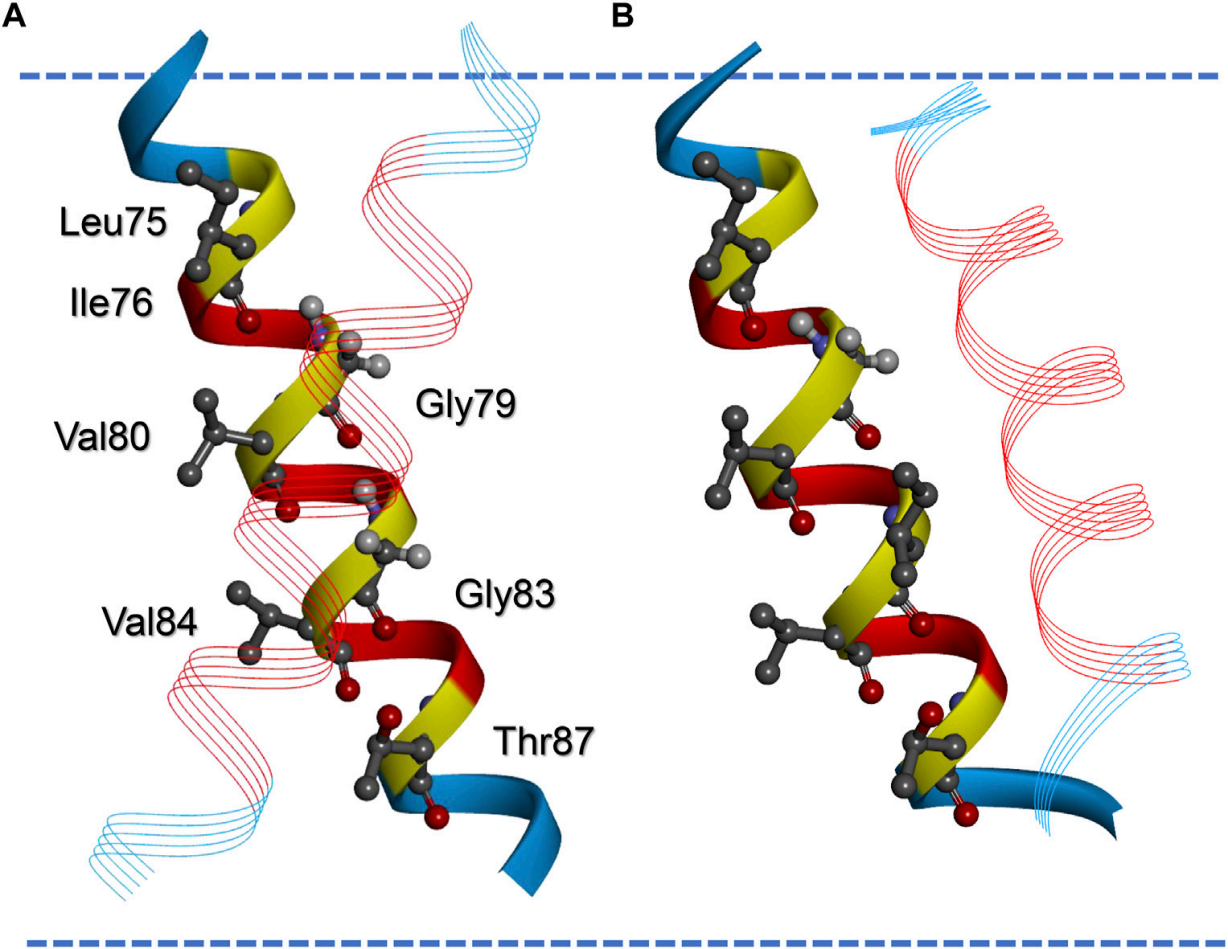
Modelled structures of the wt and G83I-mutated GpA13 TM helix dimer. Model of the wt **(A)** and the G83I-mutated GpA TM helix dimer **(B)** calculated by TMDOCK (Lomize and Pogozheva, 2017). The helices in the back are matched in orientation to allow comparison of the position of the second helix in the dimer (line ribbon presentation). Residues originating from the original GpA TM sequence are shown in red, the flanking amino acids, which were added by the expression plasmid but also contribute to TM helix formation, are shown blue. Amino acids of the GpA LIxxGVxxGVxxT dimerization motif are highlighted. Note that G83 is replaced by an Ile in **(B)**. The margins of the hydrophobic part of the membrane as calculated by the software implemented in PP3 (Lomize et al., 2022) are indicated by blue broken lines. Note that according to this model the not Leu-prolonged dimers experience negative hydrophobic mismatch, as discussed in the text.

The data shown in Figure 1 demonstrate that addition of Leu residues severely influences the interaction propensities determined in the *E. coli* inner membrane using the TOXCAT assay. However, the change did not follow the pattern expected from prolonging the GpA TM and by this just altering the level of hydrophobic mismatch between the TM and the membrane. The mean thickness of the *E. coli* plasma membrane is 37.5 Å (Mitra et al., 2004), the hydrophobic thickness is around 30 Å, as estimated based on the TM protein data base OPM (Lomize et al., 2006). The calculated hydrophobic TM length of the GpA-derived part of the GpA13 construct (LIIFGVMAGVIGT) is 19.5 Å and increases to about 30 Å upon addition of seven Leu residues, assuming an increase of 1.5 Å/residue. Thus, the construct originally defined as the minimal TM required for proper dimerization in the ToxR-assay (Langosch et al., 1996) is expected to experience severe hydrophobic mismatch, and with seven added Leu residues, the monomeric TM would just reach the hydrophobic thickness of an *E. coli* membrane. Since in the dimer the individual TM helices are tilted (crossing angle about 42°, (MacKenzie et al., 1997; Mineev et al., 2011; Trenker et al., 2015)), the hydrophobic thickness is further reduced by about 7%. Thus, considering only the GpA TM part of the sequence, a continuous decrease of the hydrophobic mismatch would occur upon prolongation with Leu residues. Yet, it is difficult to reconcile the pattern of the observed CAT activities with this prediction. However, when looking at the flanking amino acids in the construct, we suspected that the actual TM helix of the GpA13 reference structure might be longer than expected when considering solely the GpA TM helix part. Strikingly, the C-terminal next three residues (ILI) are very similar to the ones found in the GpA sequence (ILL). The three amino acids at the N-terminal end could also be part of a TM helix (RAS, Figure 1A). In order to estimate the TM helix length of the GpA13 fusion construct, a model of the TM helix was generated using the program FMAP (Lomize et al., 2011). This tool allows to determine the putative length of a TM helix within a longer stretch of amino acids. Indeed, the resulting model has a TM helix which is considerably longer than the 13 aa of the GpA TM fragment, involving the preceding and following three amino acids in the sequence. According to this model, the hydrophobic thickness of the monomeric TM is approximately 28.5 Å. The corresponding dimer in an *E. coli* membrane, modelled by TMDOCK (Lomize and Pogozheva, 2017) and PP3 (Lomize et *al.*, 2022) shows the typical GpA dimer structure (Figure 3). The crossing angle of the helices in this model is 44°, in excellent agreement to the one experimentally observed in the x-ray structure of GpA_M^81^I [42°, 5EH4. pdb, (Trenker et al., 2015)]. Nevertheless, the thickness of the hydrophobic core of the predicted GpA13 dimer still is shorter than the hydrophobic thickness of the membrane, yet it is rather close to it. For the GpA_G83I mutant, the overall structure of the modelled dimer differs substantially from the wt: the predicted helix crossing angle is smaller (38°), and TM helix-helix interactions involve different residues. Compared to the wt, where Gly79 and Gly83 are identified being key for interaction, for GpA_G83I the residues Phe78, Ala82, and Gly86 line the interaction surface (Figure 3).

Thus, when the flanking residues introduced by the expression plasmid are considered and added to the GpA13 TM sequence, addition of 2–3 extra residues are sufficient to roughly match the hydrophobic thickness of the *E. coli* membrane. Thus, the abrupt change experimentally observed in the dimerization propensity for the wt constructs would (roughly) correspond to the length were positive hydrophobic mismatch starts to occur. Indeed, in a MD simulation (Zhang and Lazaridis, 2009), the flanking amino acids added to the TM due to the construction of the TOXCAT fusion protein were identified to crucially modulate the GpA TM helix dimerization propensity. Also, a particular role of the Arg introduced in this construct was discussed, which is located at the same position as in the GpA construct employed in the present study (Figure 1A). These observations support our hypothesis that the here observed marked decrease in the CAT activity in case of the wt data marks the onset of positive hydrophobic mismatch caused by prolongation of the TM with Leu residues.

Positive hydrophobic mismatch, resulting from an increased length of the hydrophobic region of a TM peptide relative to the membrane core region, results in insufficient shielding of the hydrophobic residues of the TM helix. This might induce additional structural adaptations of the helix and/or membrane structure (Killian, 1998), possibly leading to membrane deformation (Petrache et al., 2000; Mitra et al., 2004; Grau et al., 2017) and/or to unspecific protein aggregation (Sparr et al., 2005). Aggregates are expected to have an oligomer structure differing from the classical GpA dimer structure, thus being (largely) independent on the exact sequence. In fact, the dimerization level obtained for the wt and mutant construct was similar when more than one Leu was added to the GpA13 TM helix. Thus, possibly the here analyzed TM helices can be divided into two classes: 1) (close to) matching conditions, up to one added Leu residues, where both wt and mutant constructs show defined, but different dimer structures, and 2), conditions of hydrophobic mismatch when more than one Leu were added and sequence-unspecific aggregates formed. Clearly, TM helix-helix interactions measured within the *E. coli* inner membrane depend 1) on sequence-specific interactions as well as 2) on hydrophobic (mis)match and both have to be considered when interaction propensities are measured using genetic systems.

It cannot finally be resolved, why addition of just one further Leu residue apparently promotes the prompt transition from a sequence-specific interaction to unspecific aggregation. One would not expect a complete loss of specific dimerization upon addition of one further Leu residue just due to increased hydrophobic mismatch. Thus, other structural adjustments of the dimer structure upon changing the hydrophobic (mis)match, such as changes in the amino acids included in TM helix formation, or the helix crossing or tilt angle in the dimer are likely to contribute. Furthermore, upon addition of residues at exclusively one TM end, the position of the central GxxxG motif within in the membrane might be shifted, which might be energetically unfavorable, as discussed in (Zhang and Lazaridis, 2009). Interestingly, when adding Leu residues on both ends of a short TM helix, the dimerization propensity was not altered dramatically in a biological assay similar to TOXCAT (Grau et al., 2017). Furthermore, differences in the effect of the Leu addition in different cell lines were observed, again indicating the presence of multiple adaptation mechanisms in response to hydrophobic mismatch.

Possibly, synergistic effects between different levels of structural adjustments occur, similar to what has been found in double Ala mutation studies of the GpA TM helix, where clearly strong synergistic effects were observed between substitutions at each end of the TM helix, namely between Leu75 and Thr87 (Doura and Fleming, 2004). This study suggests that apparent slight changes in TM sequence and/or length might have a large impact on its dimerization propensity due to still unknown synergistic mechanisms, in particular when employing a biological read-out such as the TOXCAT assay, which relies on proper dimerization of the attached ToxR domains.

In contrast, when studying dimerization of GpA TM peptides in artificial membranes, no such sharp change in dimerization propensity upon onset of positive hydrophobic mismatch has been observed (Anbazhagan and Schneider, 2010). Thus, it seems that the repertoire of adaptations to hydrophobic mismatch is larger in biological membranes than in artificial ones, supported by the observation that membrane thickness of biological membranes clearly changes upon removal of TM proteins (Mitra et al., 2004). Thus, the sharp drop observed in case of the wt might be the result of multiple processes, which are not yet completely understood, possibly also involving membrane crowding, a currently little explored aspect (Loewe et al., 2020). The immediate lipid environment surrounding a TM protein, *e.g.* the presence of particular lipids, can also modulate the interaction propensity. For example, it has been hypothesized that the avoidance of local membrane structure perturbations in the vicinity of membrane proteins [“lipophobic effect,” Duneau et al., 2017)] can trigger dimerization of membrane proteins. The level of such perturbations could depend on helix length and/or the level of hydrophobic mismatch, even for the same membrane. Furthermore, a specific interaction of the GpA TM helix with defined lipid species has been shown to modulate the GpA dimerization propensity (Hong and Bowie, 2011). While the lipid environment remains unchanged when GpA TM helices with different length were analyzed in the present study, changes in the TM topology, such as the helix tilt angle, can clearly have an impact on the strength of a given protein-lipid interaction, resulting in altered determined interaction propensities. Finally, in genetic assays based on analyses within cellular membranes clearly an analyzed TM helix has to be sufficiently long to properly integrate into the biological membrane. Thus, in contrast to micellar systems, *in vivo* systems the thickness of the biological membrane sets (at least) a lower limit for the length of a TM helix that can still be studied using genetic systems, such as TOXCAT.

## Conclusion

The here described results illustrate that interaction propensities determined within the *E. coli* inner membrane using genetic assays, such as the TOXCAT assay, can strongly depend on the length of a given TM helix. Furthermore, when interpreting the results, it might be necessary to include the amino acids flanking the actual TM part. While this probably is less relevant when variants of a single TM helix are compared and analyzed, and here the impact of single amino acids on a TM helix oligomer structure can be deduced, it clearly has to be considered when different TM helices are compared. Nevertheless, as observed here, also in case of variants of a single TM helix, the analyzed TM helix length matters. In many studies a crucial involvement of Gly83 in GpA TM helix dimerization has been described (Lemmon et al., 1992; Russ and Engelman, 1999; Fleming and Engelman, 2001; Schneider and Engelman, 2004b), yet, when the GpA wt and G83I mutated TM helices with ≥2 added Leu residues are compared, a crucial involvement of Gly83 cannot be deduced from the experimental results.

Thus, while genetic assays have clearly helped to study and better understand TM helix-helix interactions in the recent decades, results obtained with such systems must be interpreted with great care, and the length of the analyzed helices must always be considered when a study involving such systems is designed and/or the results are compared.

## Data Availability

The original contributions presented in the study are included in the article/Supplementary Material, further inquiries can be directed to the corresponding author.

## Abbreviations

CAT, chloramphenicol acetyl transferase; GpA, glycophorin A; MalE, maltose binding protein (of *E. coli*); MD, molecular dynamics; TM, transmembrane; wt, wild type.
